# Schema-driven prediction effects on episodic memory across the lifespan

**DOI:** 10.1098/rstb.2023.0401

**Published:** 2024-09-16

**Authors:** Javier Ortiz-Tudela, Gözem Turan, Martina Vilas, Lucia Melloni, Yee Lee Shing

**Affiliations:** ^1^ Mind, Brain, and Behavior Research Center, Department of Experimental Psychology, University of Granada, Granada, Spain; ^2^ Department of Psychology, Goethe University Frankfurt, Frankfurt, Germany; ^3^ Center for Individual Development and Adaptive Education of Children at Risk (IDeA), Frankfurt, Germany; ^4^ Research Group Neural Circuits, Consciousness and Cognition, Max-Planck Institute for Empirical Aesthetics, Frankfurt, Germany

**Keywords:** schemas, predictive processing, episodic memory, lifespan

## Abstract

The predictive processing framework posits that one of the main functions of the brain is to anticipate the incoming information. Internal models facilitate interactions with the world by predicting future states against which actual evidence is compared. The difference between predicted and actual states, the prediction error (PE), signals novel information. However, how PE affects cognitive processing downstream is not fully understood: one such aspect pertains to how PE influences episodic memories, and whether those effect on memory differ across the lifespan. We examine the relationship between PE and episodic memory in children, young and older adults. We use a novel paradigm whereby rich visual narratives are used to build action schemas that enable probing different mnemonic aspects. To create different levels of PE, we manipulate the story endings to be either expected, neutral or unexpected with respect to the unfolded action. We show that (i) expected endings are better encoded than neutral endings and (ii) unexpected endings improve the encoding of mismatching events and other aspects of the narrative. These effects are differentially modulated across the lifespan with PE-driven encoding being more prominent in children and young adults and with schema integration playing a larger role on memory encoding in older adults. These results highlight the role of predictions by enriching past experiences and informing future anticipations.

This article is part of the theme issue ‘Elements of episodic memory: lessons from 40 years of research’.

## 1. Schema-driven prediction effects on episodic memory

Prediction is a core cognitive operation that underpins our ability to anticipate and prepare for future events. It facilitates not only the execution of everyday tasks, such as catching a ball or routing through traffic, but also informs complex decision making and planning. This anticipatory mechanism is central to adaptive behaviour, allowing individuals to navigate their environment by mentally projecting possible future scenarios. Doing so helps our brains to prepare for what comes next, bridging the gap between present experience and future events, and thus eases our interactions with the world. Although different types of predictions might be required for different goals (e.g. catching a ball versus planning a trip versus other; [1]), anticipating any kind of future states largely relies on memory. Mnemonic traces provide the building blocks upon which predictions are formed. In recent years, the bidirectional relationship between memory and predictive processes has increasingly become the subject of research [2–11].

A critical component of predictive processing is the monitoring of discrepancies between expected and actual outcomes (i.e. prediction errors, PE). These errors highlight novel information unaccounted for by existing knowledge, signalling the potential need for updates of the brain’s internal model thereby triggering learning. The integration of this new information is thus crucial for refining future predictions. Various mechanisms have been proposed to explain how the brain incorporates PE-related information, with all converging on the hypothesis that the encoding of information is enhanced following unexpected events in comparison to expected ones [6,12–14].

Simultaneously, reinforcing accurate predictions by learning from them ensures that reliable associations are strengthened, thus enhancing efficiency and accuracy in our interactions with highly predictable environments. In line with this, a wealth of studies supports the beneficial mnemonic effects of information that is predictable and/or congruent to prior knowledge [15–18]. Recent theoretical advances suggest that the encoding of information in the brain is driven by two opposing mechanisms whose relative contribution is determined by the accuracy of the prediction. On the one hand, predictions that align with actual events reinforce existing associations thereby strengthening previous models; on the other hand, strong PEs prompt the formation of new memory traces and/or the updating of current models to accommodate the unexpected information. Critically, these proposals postulate that in intermediate situations where the received information is neither fully predictable nor entirely surprising, none of these encoding boosting mechanisms exert a strong influence on memory. Consequently, the mnemonic encoding of these situations would be lowest, thus rendering a *U*-shape relationship between the strength of PE and memory performance [5,11,19], such that memory encoding is best at lowest and highest levels of PE and minimal at intermediate levels.

Different levels of PE strength can be estimated by manipulating the degree to which a given event matches the current contextual schema. Schemas, understood as cognitive frameworks constructed from the abstraction of multiple interactions with the environment, can act as the source for the anticipation of future events [20]. Recent human behavioural and neuroimaging results suggest that contextual schemas allow people to be selective in when they encode and retrieve episodic memories, indicating that encoding and retrieval are influenced by uncertainty about upcoming states and the risk of retrieving irrelevant memories [21]. Critically, these schemas develop and transform throughout the lifespan [22]. In childhood, schemas do not (yet) represent a stable and accurate model of the environment and children need to learn to assimilate new experiences rapidly. As we progress into adulthood, these cognitive models become more elaborate and stable, allowing for more nuanced predictions based on a wider array of experiences across contexts. However, this increased precision might come at the cost of reduced flexibility of these schemas, potentially leading to difficulties in updating in the face of novel or changing information. We postulate that as a consequence of such age-related differential exploitation of underlying schemas, the impact of PEs on memory encoding is probably to also exhibit notable variations across the lifespan. This notion is compatible with recent findings, demonstrating that the dynamics of episodic memory encoding and retrieval are heavily influenced by the evolving nature of schemas [21] and are thus probably to be differentially affected by the schema-driven PEs.

Moreover, the impact of PE on memory is also probably not to be uniform but rather might differentially weight various memory features. Indeed, episodic memories are intrinsically rich, multifaceted traces containing different information content (e.g. actors involved in a scene, actions being performed, physical and temporal context, etc. [23–26]). Previous studies have found that PE can selectively affect which contents will be encoded and remembered. For instance, PE can enhance the encoding of specific characteristics of the actors in a scene (e.g. which cloth they wore [27]), but it does not increase memory for what action they were engaged in before the PE [28].

## 2. The present study

The current online study examined lifespan differences in the use of schemas to generate episodic memories with the goal of dissociating the aforementioned encoding mechanisms. To achieve this, we utilized visual narratives in the form of comic strips, comprising a series of panels that depict an action unravelling over time. On the one hand, this format combines the advantages of discrete presentation with a temporal element, allowing for the controlled unfolding of events akin to videos, yet with greater precision in timing control [28]. On the other hand, unlike static images, these visual narratives naturally prompt the anticipation of future events, making them an ideal tool to manipulate PE and schema congruence. Additionally, visual narratives allow the use of content-rich and outside-the-lab-like stimuli which fosters engagement particularly at both ends of the lifespan continuum.

**Figure 1.**
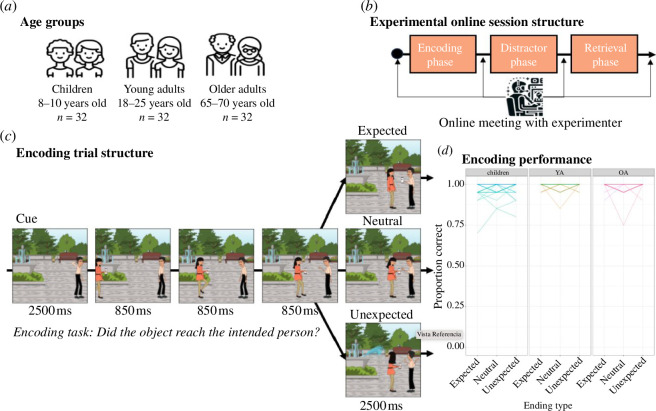
Session structure and encoding phase. (*a*) Three age groups participated in the study. (*b*) Structure of the experimental session comprising an encoding phase, a distractor phase and a retrieval phase, conducted during an online meeting with the experimenter. (*c*) Trial structure for the encoding task. Three different ending types were included. (*d*) Encoding performance across age groups. Each line represents one participant with thicker lines resulting from overlapping (mostly ceiling) performance.

Participants from three age groups (figure 1*a*)—children, young adults and older adults—met with the experimenter in an online virtual room and conducted the entire session in a one-to-one setting akin to that of traditional in-lab experiments (see more on this below). During these online meetings (figure 1*b*), participants were exposed to multiple comic strips (i.e. encoding phase) and later their memory about four different aspects of the events was assessed (i.e. retrieval phase). These different memory tests aimed to evaluate the degree of familiarity with the comic in addition to details present in the scenes conveying both central and superficial information. Specifically, we first tested for the overall memory of the visual narrative. Here, we presented the first image of the series which did not contain any information about the action or the ending of the story (cue recognition). This was done to prompt retrieval based on peripheral details such as the clothes of the characters or the physical context. Second, we assessed memory retrieval of central elements of the narrative (object recall). Third, we tested the participant’s memory of the feeling of closure of the event (outcome memory) irrespective of the specific content (e.g. ‘I remember something unexpected happened but not what’). Finally, we assess the memory retrieval of the specific ending of the narrative when the PE took place (ending type). In line with the pre-registration, we hypothesized that in children, the encoding mechanism sensitive to PEs would play a dominant role due to their developing, yet incomplete schemas. This should result in enhanced memory encoding for unexpected information, but less so for schema-congruent information (i.e. left-tilted *U*-shape relation). Conversely, in older adults, we anticipate the opposite pattern. Due to more established schemas, their encoding process is hypothesized to favour schema-congruent information, demonstrating a reduced sensitivity to PEs (i.e. a right-tilted *U*-shape). Young adults, situated at an intermediate stage of lifespan cognition, are expected to exhibit both mechanisms operating at their peak. Together, the hypothesized patterns of results aim to illuminate the developmental trajectory of the postulated encoding mechanisms and provide a clear double dissociation between the enhancement effects of PEs and the reinforcement of schema-congruent encoding.

## 3. Methods

### (a) Participants

Children (8–10 years old), young adults (18–25 years old) and older adults (65–70 years old) were recruited from the lab participant database or the local community through flyers and posters. Efforts were made to achieve a balanced number of male and female participants whenever possible. Participants were fluent speakers of either German or English, and no other restrictions were applied for recruitment. All participants received monetary compensation for their participation and provided informed consent before participating in the study. In the case of minors, parents or legal guardians provided informed consent, and minors gave assent. The study protocol was approved by the ethics board of Goethe University Frankfurt.

To estimate the targeted sample size, the WebPower library in R was used to conduct an *a priori* power analysis for a repeated measures analysis of variance (ANOVA) with one within-participants factor having three levels (expected, neutral and unexpected) and one between-participants factor also with three levels (children, younger adults and older adults). The aim was to achieve an 80% power to detect a minimum effect of 0.25 (Cohen’s *f*) at the standard alpha level of 0.05. The estimated sample size (*n* = 193.36) was adjusted to be evenly divisible by the number of groups, resulting in a total of 192 (*n* = 64 per age group), leading to an effective power of 79.7%.

A sequential analysis approach was adopted by Lakens [29]. This involved including one interim analysis step when half of the sample was collected (*n* = 32 per group; step size 32 × 3 = 96 participants). The GroupSeq library in R was used to adjust the critical alpha values for each analysis step while maintaining the overall alpha level at 0.05. We used the O’Brien–Fleming procedure [30] which applies a stricter correction to the alpha level at the initial interim steps when variability is expected to be high. This stricter criterion is then relaxed so that the alpha level for the final step remains close to the standard level of 0.05. Following this procedure, if the main effect of ending type in the memory tasks was statistically significant at the first interim step (with an alpha threshold of 0.003), data collection would be terminated. Similarly, data collection would also cease at the first interim step if the observed effect sizes were smaller than 0.1 (Cohen’s *f*). If neither condition was met, data collection would proceed until the maximum sample size was achieved, using an alpha level of 0.047 to declare significance.

The interim analysis step revealed that the main effect of ending type for both the cue and ending recognition was significant at *p* < 0.003. In contrast, for the object recall task, the effect size was very small (*η*^2^*G* = 0.006), suggesting the lack of an effect which would not be compensated by continued data collection. Finally, for the outcome memory, only young adults performed above chance, so we just explored the effect of ending type in this group. The main effect was *p* = 0.004 which we deemed close enough to 0.003 to justify halting data collection in the context of all four analyses. The final sample comprised 32 children (mean age = 9.5 years, s.d. = 3.4, 18 females), 32 young adults (mean age = 22.3 years, s.d. = 2.1, 24 females) and 32 older adults (mean age = 67.5 years, s.d. = 2.6, 16 female).

### (b) Stimuli

The stimuli used consisted of 80 comic strips, each comprising five panels (figure 1*c*). Individual comic strips were screened for arousing stimuli (e.g. spiders) and replaced with more neutral ones. For each participant, 60 strips were used in the encoding phase and the other 20 were reserved to be used as foils in the memory phase. Specific strips were counterbalanced across participants so that all stimuli served as foils throughout the entire sample. Each strip depicted two characters engaged in a simple action (i.e. throwing or serving an object) that unfolded across the five panels (figure 1*c*). The outcome of the actions in the last panel was manipulated to create three distinct ending conditions, each represented with 20 comic strips. The first condition was the ‘expected ending’ (figure 1*c*, top panel), where the action concluded as anticipated (i.e. one of the characters successfully receive the intended object). The second condition, the ‘unexpected ending’ (figure 1*c*, bottom panel), introduced an element altering the course of the narrative and thus causing the anticipated action to be interrupted. The third condition was the ‘neutral ending’ (figure 1*c*, middle panel), where the narrative sequence was left incomplete, with the action’s resolution being ambiguous. Unlike the other conditions where the last panel revealed the action’s resolution, this condition used a continuation frame in the sequence—such as a ball that is still in motion towards the second character but not yet caught. This ending condition was used as an intermediate situation where the anticipated action is not matched but also not mismatched, thus enabling a scenario where memory encoding would not benefit from schema integration or from PE.

### (c) Design and procedure

The study employed a 3 (ending type: expected, neutral or unexpected) by 3 (age group: children, younger adults or older adults) mixed design. The ending type was manipulated within participants, with each participant experiencing all three ending conditions. On each session, participants carried out an encoding phase, followed by a distractor phase in which they performed math operations for 10 min and finally a retrieval phase (figure 1*b*).

All testing was carried out online. Stimulus presentation and response collection were programmed in PsychoPy v. 2021.1.4 and hosted on Pavlovia (https://pavlovia.org). At the beginning of the testing session, the experimenter met the participant in a virtual room using an online videoconferencing tool (figure 1*b*). During this initial meeting, the experimenter assessed the appropriateness of the testing setup through a brief set of questions. These questions focused on the participant’s overall wellbeing, the physical environment of the room where the task would be performed and the computer being used for the session. All participants were instructed to sit in a quiet room, using a laptop or a desktop computer, and were encouraged to minimize distractions as much as possible throughout the session. In addition, verbal instructions about the tasks were provided before each phase by the experimenter and participants had the opportunity to ask questions. At the start of each phase, instructions were again presented in written format. Note that the memory test was not mentioned before the memory phase. At the conclusion of the session, the experimenter reconnected with the participant through the videoconferencing tool to inquire about any unforeseen events or situations that might have occurred during the completion of the task.

### (d) Encoding phase

During the encoding phase, participants were presented with 60 comic strips in a randomized order, determined by PsychoPy’s internal routines. Nine additional comic strips (i.e. three of each ending condition) were included at the beginning as practice trials and not used further. Each trial (figure 1*c*) began with a fixation cross displayed for 0.5 s, followed by the presentation of the cue (i.e. the first panel of the comic strip) for 2.5 s. The first panel in the sequence contained a single character in a unique context which served to situate the context of the story but provided no information with respect to the action that would unfold over the sequence (i.e. a serve or a throw action), nor of the object and second character that initiated the action. This was succeeded by the action sequence, where across three subsequent panels the action unfolded (comic strip images). Each image of the comic strip was displayed for 0.85 s. The trial concluded with the target (i.e. the last panel of the comic strip) displayed for 2.5 s. Each comic strip included one of the three possible ending types: an expected, unexpected and a neutral ending. In the expected condition, the final panel was congruent with the narrative schema developed in the preceding panels. In the unexpected condition, the final panel was incongruent with the unfolding schema. In the neutral condition, the final panel depicted an instance of the action that remained unresolved from the previous slides, leaving the narrative’s conclusion ambiguous. Each ending was presented in equal proportion for a total of 20 trials per condition. On every trial, participants were required to use two keys on a keyboard to report as accurately as possible on the outcome of the actions depicted (i.e. whether the object reached its intended recipient). Speed was not stressed.

### (e) Retrieval phase

**Figure 2.**
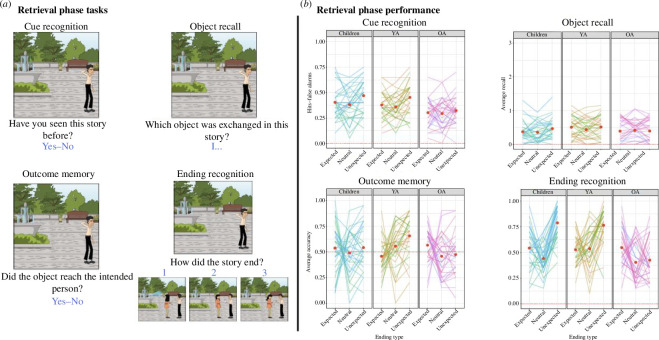
Retrieval phase. (*a*) Tasks involved in the retrieval phase included cue recognition, object recall, outcome memory and ending recognition. (*b*) Performance across the three age groups for each task depicted in (*a*).

The retrieval phase assessed participants’ memory of the comic strips presented during the encoding phase. This phase consisted of four different memory tasks (figure 2*a*), namely, cue recognition, object recall, outcome memory and ending recognition, each designed to probe different aspects of memory of the comic strips. Each trial began with a fixation cross followed by a series of tasks presented in sequence; for these tasks, stimuli remained on the screen until the participant responded.

#### (i) Cue recognition

In the first task, participants were presented with the first panel of a comic strip which served as a retrieval cue of the episode. Note that this cue image depicted only one character and no information about the action, nor the object involved in the action. Cues for all 60 previously presented comic strips belonging to the three different ending conditions, i.e. expected, unexpected and neutral endings were presented. Those were intermixed with 20 fillers images that were never seen before. Cues were presented in random order. For each cue, participants judged the images as old or new (i.e. ‘Have you seen this story before?’) (figure 2, top left).

#### (ii) Object recall

In the second task, while the cue image remained on the screen participants were asked to recall the central object featured in the comic strip (i.e. ‘Which object was exchanged in this story?’). They were instructed to type on the keyboard a maximum of three words describing the object (e.g. red soda can) and to press ENTER to submit their response. Responses were later manually coded by a naive rater as follows: 0 = the response was incorrect; 1 = the response matched the action performed; 2 = the response matched the type of object in the sequence; and 3 = the response matched the actual object in the sequence. The coder did not have access to the actual ending or to its type but only to the first slide in which the object appeared and to a text label describing the object (figure 2*a*, top right).

#### (iii) Outcome memory

After the recall responses had been submitted, they disappeared from the screen and the third task was presented. With the cue image still displayed on the screen, participants were instructed to report the outcome of the action that unravelled in that story (i.e. ‘Did the object reach the intended person?’) by pressing one of two keys on the keyboard (figure 2*a*, bottom left).

#### (iv) Ending recognition

Finally, for the fourth task, three alternatives depicting the three possible endings, expected, neutral and unexpected, were presented at the bottom of the screen with the cue still displayed at the top. One image corresponded to the ending of the comic strip previous seen by the participant while the other two images acted as lures. Participants were instructed to select the ending that they had seen during encoding (figure 2*a*, bottom right).

The use of four distinct memory tasks within the retrieval phase aimed to provide a comprehensive evaluation of the episodic traces generated during encoding. Each task taps into different mnemonic aspects thus enabling the assessment of not just whether an event is remembered, but also how various elements of that event might be differentially impacted by PE across the lifespan. The multidimensional nature of episodic memory makes this approach key to reconcile existing findings and conflicting accounts.

### (f) Deviations from the registered protocol

A pre-registration was created and is available at [31]. Note that a few datasets (*n* = 11) were collected but not analysed before the freezing of the registration due to logistic issues. The only deviation from the pre-registered analysis plan is included below.

The registered analysis plan included an exclusion criterion based on performance on the cue recognition task to control for lack of task engagement during the second half of the session. However, this criterion would exclude 19 participants, who otherwise showed above-chance performance in the other memory tasks indicating that subject’s performance across tasks was indeed reliable. Therefore, considering the high performance in the encoding task and also the relatively high performance in the other three memory tasks, we opted to forgo the exclusion criteria in the final analysis. The results are however robust to the exclusion criteria applied (refer to electronic supplementary materials for the results with the preregistered exclusion criteria).

### (g) Statistical analysis

For each task, overall performance was evaluated against chance level in R with the libraries *rstatix* and *BayesFactor.* Note that this was not done for the object recall task as it is not straightforward to define the chance level in this task.

As stated in our pre-registration, we took an ANOVA approach to analyse our data and complemented it with a linear mixed model (LMM) approach. As both sets of analysis rendered similar results, and we deemed the LMM approach as more flexible in being able to model trial-level data, we report the outputs from this approach (refer to electronic supplementary materials for the ANOVA results).

To determine the best-fitting model for our analysis, we used a backward selection method [32,33]. We began with the most complex model that included age group, ending type and their interaction as fixed effects, and independent slopes and intercepts for participants and comic strips as random effects. From this full model, we systematically removed random parameters at a time to create a series of simpler models. Each time, we compared the complex model with the next simplest model using a likelihood ratio test [34]. If removing a parameter did not significantly worsen the model fit (*p* > 0.05), we adopted the simpler model. This process was repeated until removing any further parameters significantly reduced the model’s explanatory power. We then performed Wald chi-squared tests to assess the significance of the fixed effects. For the sake of simplicity, only the final winning models are reported here but the complete analysis pipeline and dataset [35] to reproduce this procedure is available at [36]. Once we identified the final model, we used contrasts to test for the presence of linear and quadratic components of the ending type effect on memory performance.

## 4. Results

### (a) Encoding phase

Participants’ responses during encoding (figure 1*d*) showed overall accuracy reaching almost ceiling performance for children (mean accuracy = 0.97, s.d. = 0.21), young adults (mean accuracy = 0.99, s.d. = 0.08) and older adults (mean accuracy = 0.99, s.d. = 0.14). A Kruskal–Wallis test with group as a between-participants factor revealed a significant effect of group, *H*(2) = 19.3, *p* < 0.001. Follow-up pairwise comparisons using the Mann–Whitney *U*-test showed significant differences in accuracy between children and older adults (*U* = 3682, *p* = 0.003) and between children and young adults (*U* = 3568, *p* < 0.001), with children performing lower than both older adults and young adults. No significant difference in accuracy was observed between young and older adults (*U* = 4504, *p* = 1.000). Despite these minor differences (2%), the analysis of the accuracy during encoding indicated almost ceiling performance and comparable engagement with the task in all age groups.

### (b) Retrieval phase

#### (i) Cue recognition

Overall *d*’ was computed for every participant from hits and false alarm rates and tested to be different from zero, *t*(95) = 24.233, *p* < 0.001, Cohen’s *d* = 2.47, BF_10_ = 1.106 × 10^39^ (children *d*’ = 1.26, young adults *d*’ = 1.07 and older adults *d*’ = 0.822, all individual *p*s < 0.001). Since it is not possible to compute independent false alarm rates for each ending type condition, the proportion of hits was submitted to our modelling procedure to test for effects of interest.

**Figure 3.**
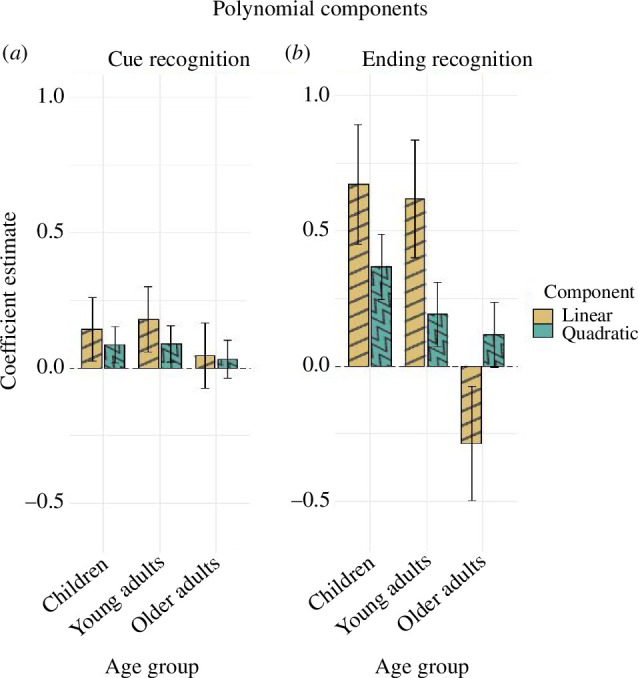
Polynomial analysis of recognition memory performance by PE across age groups. Coefficient for linear and quadratic components were estimated for (*a*) cue and (*b*) ending recognition. Error bars display confidence intervals calculated using the standard error and the critical value alpha of 0.05. Note that object recall and outcome memory results are not depicted because they did not include meaningful polynomial comparisons across age groups.

In the selected model, ending type and group were treated as fixed effects, and random intercepts were included for both participant and comic strip (BIC winner: 7478.1, reduced: 7543.0). The analysis of this model revealed a significant main effect of ending type (*χ*^2^(2) = 24.095, *p* < 0.001), a significant main effect of group (*χ*^2^(2) = 10.960, *p* = 0.004) and a barely significant interaction between group and ending type (*χ*^2^(4) = 9.500, *p* = 0.050; figure 2*b*, top left). We then characterized the relationship between ending type and memory by exploring the contributions of linear and quadratic components in each group (figure 3*a*). In the children group, we observed that the relationship between PE and cue recognition was better characterized by the combination of a linear and a quadratic component (linear component: *β* = 0.143, *z* = 2.419, *p* = 0.016; quadratic component: *β* = 0.085, *z* = 2.491, *p* = 0.013). In line with our hypotheses, the children group exhibited the best memory for unexpected endings, followed by expected ones and with worst memory for neutral ending (i.e. left-tilted *U*-shape). Similarly, in the young adults group, the linear component was stronger than the quadratic (linear component: *β* = 0.180, *z* = 2.958, *p* = 0.003; quadratic component: *β* = 0.088, *z* = 2.557, *p* = 0.011), thus suggesting a stronger influence of the PE benefit than of a schema integration mechanism. Direct comparison between children and young adults on their linear and quadratic components rendered no significant differences (linear: *β* = 0.037, *z* = 0.434, *p* = 0.665; quadratic: *β* = 0.004, *z* = 0.074, *p* = 0.941, respectively). Finally, for the older adults group, neither linear nor quadratic components were statistically significant (linear component: *β* = 0.046, *z* = 0.735, *p* = 0.462; quadratic component: *β* = 0.032, *z* = 0.903, *p* = 0.367), thus suggesting no influence of PE on recognition memory for the cue. Despite the lack of significant components, direct comparisons between children and young adults, revealed no significant differences in the linear and quadratic components (against children, linear: *β* = −0.097, *z* = −1.136, *p* = 0.256; quadratic: *β* = −0.052, *z* = −1.069, *p* = 0.285. Against young adults, linear: *β* = −0.134, *z* = −1.543, *p* = 0.123; quadratic: *β* = −0.056, *z* = −1.132, *p* = 0.258).

#### (ii) Object recall

The average rated accuracy (0–3) for the recall task was submitted to our modelling procedure. The winning model included ending type, group and their interaction as fixed effects and random intercepts for participant and comic strip (BIC winner: 13 617, BIC reduced: 13 939). Neither of the main effects nor the interaction were statistically significant—age group: *χ*^2^(2) = 2.469, *p* = 0.291; ending type: *χ*^2^(2) = 5.531, *p* = 0.063; and interaction: *χ*^2^(4) = 6.305, *p* = 0.178. The overall poor performance in this task (figure 2*b*, top right) might have prevented any boosting effects arising from different levels of PE.

#### (iii) Outcome memory

Three independent one-sample one-tailed *t*-tests against a chance level of 0.5 were conducted across age groups. Both for older adults and for children, performance did not differ from chance, *t*(31) = 0.139, *p* = 0.891, Cohen’s *d* = −0.025, BF_10_ = 0.191 (mean accuracy = 0.498) and *t*(31) = 1.646, *p* = 0.110, Cohen’s *d* = 0.291, BF_10_ = 0.634 (mean accuracy = 0.522), respectively. In contrast, young adults showed significantly higher than chance performance, *t*(31) = 3.880, *p* < 0.001, Cohen’s *d* = 0.686, BF_10_ = 59.341 (mean accuracy = 0.555).

To further explore this result, young adults’ responses were submitted to our modelling procedure. The winning model included ending type as fixed effect, and random slope and intercept for comic strip (BIC winner: 2474.7, reduced: 2481.6). This analysis revealed a significant effect of ending type, *χ*^2^(2) = 16.556, *p* < 0.001, with this effect being characterized by a positive linear component, *β* = 0.469, *z* = 3.329, *p* < 0.001 and no quadratic component, *β* = 0.020, *z* = 0.323, *p* = 0.747. In other words, young adults showed better memory for the outcome of the event with increasing PE (figure 2*b*, bottom left).

#### (iv) Ending recognition

Responses to the ending recognition task were submitted to three independent one-sample one-tailed *t*-tests against a chance level of 0.33 to validate overall performance. The results indicated significant deviations from the chance level in all groups (children’s mean accuracy = 0.589, *t*(31) = 13.599, *p* < 0.001, Cohen’s *d* = 2.404, BF_10_ = 5.070 × 10^11^. ; young adults’ mean accuracy = 0.608, *t*(31) = 20.193, *p* < 0.001, Cohen’s *d* = 3.570, BF_10_ = 2.024 × 10^16^; older adults’ mean accuracy = 0.457, *t*(31) = 8.761, *p* < 0.001, Cohen’s *d* = 1.549, BF_10_ = 1.632 × 10^7^). These results suggest that all age groups performed significantly above the chance level in the task. An analysis of confusion matrices (electronic supplementary material, figure S1) revealed a non-uniform error distribution, with misattributions more common between expected and neutral than unexpected responses. Importantly, this pattern was consistent across the three age groups (refer to electronic supplementary materials for more details).

To assess the influence of ending type on recognition memory, accuracy scores were submitted to our modelling procedure. The winning model included ending type, group and their interaction as fixed effects, and random intercept and slopes for participants and comic strips (BIC winner: 7328.1, reduced: 7333.1). We observed significant main effects of ending type, *χ*^2^(2) = 56.368, *p* < 0.001, age group, *χ*^2^(2) = 20.254, *p* < 0.001 and a significant interaction between them, *χ*^2^(4) = 64.280, *p* < 0.001 (figure 2*b*, bottom right). Exploring this interaction rendered significant linear and quadratic components in both the children group (linear: *β* = 0.673, *z* = 5.943, *p* < 0.001; quadratic: *β* = 0.369, *z* = 5.985, *p* < 0.001) and the young adults group (linear: *β* = 0.618, *z* = 5.542, *p* < 0.001; quadratic: *β* = 0.192, *z* = 3.127, *p* = 0.002). Interestingly, the quadratic component in young adults was significantly reduced compared with children (*β* = −0.177, *z* = −2.075, *p* = 0.038), a pattern not observed in the linear component (*β* = −0.055, *z* = 0.357, *p* = 0.721). This selective reduction of the quadratic component indicates a smaller difference between expected and neutral endings for young adults than for children (figure 3*b*). This result is compatible with a weakening of the schema integration mechanism in young adults, which was not anticipated in our hypotheses. In sharp contrast with the other groups, the older adults showed the opposite trend in the linear component (*β* = −0.285, *z* = −2.628, *p* = 0.009), indicating worse performance as PE increases. In addition, their quadratic component was marginally significant, *β* = 0.117, *z* = 1.913, *p* = 0.056, suggesting a slight upward trend with higher levels of PE. Direct comparison of the older adults’ components with the children group revealed a decrease in the magnitude of both components (linear: *β* = 0.957, *p* < 0.001; quadratic: *β* = 0.252, *p* = 0.003). The comparison with young adults rendered a significant decrease in the linear component (*β* = 0.902, *p* < 0.001) but not in the quadratic one (*β* = 0.075, *p* = 0.377). Overall, the reduced PE benefit on and the increased memory for expected endings support our hypothesis for the older adults group, namely, a maintained schema integration mechanism in combination with a deteriorated PE-encoding mechanism would improve performance more strongly for one end of the PE continuum (i.e. right-tilted *U*-shape).

## 5. Discussion

The present study aimed to dissect the influence of anticipating events (i.e. prediction) on episodic memory encoding across the lifespan. To this end, we tested participants’ memory for comic strips with varying levels of expectedness in their endings. Our findings highlight that predictions have a multifaceted impact on the memory of varying features of experience, in which distinct age-related differences emerge across the lifespan. For both memory for the start (cue recognition) and ending of the comic strips, children exhibited an overall left-tilted *U*-shaped relationship, with memory encoding being strongest for events with highly unexpected endings, followed by a smaller memory advantage for events with highly predictable endings, in comparison to events with endings of medium PE. The hypothesized left tilt in this pattern (i.e. stronger encoding for unexpected information) aligns with the notion that developing schemas in children are particularly attuned to assimilating novel, unexpected information. The young adults group showed a similar pattern with a preference for the encoding of unexpected information. Conversely, older adults exhibited little overall benefit of PE in episodic encoding, with the opposite pattern arising for ending recognition (i.e. better memory for expectation-matching endings). This differential impact across memory tasks and age groups supports the presence of two distinct mechanisms that govern memory formation: schema integration and PE-triggered encoding. Our results showed that schema integration is more prominent in older adults’ memory, while PE-triggered encoding is more prominent in children’s memory.

### (a) Prediction (error) impacts different aspects of the encoded episode

A comprehensive view of episodic memory requires considering it as a multifaceted trace in which different features are combined to construct an episode and which can, in turn, be used to reconstruct the experience mnemonically after it has finished. This reconstruction is the key to accessing the past and, at the same time, sets the basis for anticipating the future. The capacity to simulate potential outcomes based on our extrapolations from past experiences is essential for effective decision making and adaptability in changing environments.

The relationship between episodic memory and the anticipation of future states is far from unidirectional. It has been proposed that predictive processing might have a critical role in shaping how our brains store episodic information [11,12,19,33,37,38]. In the current study, we attempted to jointly characterize the effect of expectedness on four different features of the retrieved episode.

#### (i) Cue recognition

Participants from all age groups performed a recognition memory task on the first panel of the comic strips include information neither about the ending nor about the action, the object or the second character. As such, participants’ judgements of the story could be made based on peripheral details encoded during the prediction (mis)match (e.g. remembering the t-shirt that the character was wearing when she caught the ball) or due to a retroactive influence of PE on the encoding of the beginning of the event. However, a recent study speaks against this latter mechanism. In this study, the authors showed that surprise does not retroactively enhance memory for events leading up to unexpected endings [28]. Therefore, we interpret our cue recognition task as a measure of the encoding of details of the outcome which are not directly related to the prediction (mis)match. These include the main character (e.g. their appearance) or the setting (e.g. the background location). Encoding of these details is proposed as a core feature of the snapshot-like holistic encoding of the event of the PE mechanism [5,11,39].

In line with the hypothesis that the PE mechanism is impaired with increasing age, older adults’ performance showed no significant modulation as a function of expectedness. In contrast, children and young adults predominantly exhibited a positive linear trend along the expectedness axis (i.e. enhanced memory for unexpected endings). In addition, children showed enhanced encoding of peripheral details of the outcome also for expected endings with young adults displaying a similar, though less pronounced pattern. This finding, akin to the standard memory congruency effect [16–18,40], was somewhat not anticipated for this type of information, as only the PE mechanism was hypothesized to enhance the encoding of peripheral details. However, as children’s knowledge is still being built up, remembering peripheral details during both prediction confirming and disconfirming experiences would help to stabilize knowledge while enabling flexible updating when needed. This post hoc interpretation suggests a refinement of the schema integration mechanism to also include the encoding of peripheral details depending on the robustness of the schemas. In other words, in expected situations, both central and peripheral details would be encoded if the schemas are not robust enough to deem peripheral details irrelevant (as would be the case in children or when learning in a novel context). Future studies are needed to directly test this hypothesis.

#### (ii) Object recall and outcome memory

The limited variability in the possible actions and types of objects depicted in the comic strips probably created a situation of high interference, such that memory individuation for each of the comics may have been harder, thus leading to worse performance in memory tests. Both children and older adults did not show reliable memory either for the central object exchanged in the comic strip or for the outcome of the action and no modulation of these measures by PE; young adults, however, despite their low object recall performance, showed enhanced encoding of the outcome of the story for unexpected endings. This latter result adds to the ones for story recognition by further supporting the notion of holistic encoding following PE [5,39]. It is well known that PEs trigger a myriad of cognitive and physiological responses which are immediately measurable [41–44]. These responses might be encoded into memory independently of the content of the event (e.g. I remember something unexpected happened, but I do not remember what). This was the case in our young adults group where they showed better remembering of the type of ending when these were unexpected. However, given the overall poor performance in these tasks and the modest size of the effects, we advise caution when interpreting this result. Follow-up studies with more variable sets of actions and objects might not only provide an extension of this result but also help to uncover modulations by PE which might have been obscured by floor effects.

#### (iii) Ending recognition

Our final memory task presented participants with all three potential endings, asking them to identify the one they had actually seen. This task was designed to attempt at dissociating two encoding mechanisms: schema integration and PE-driven encoding. On the one hand, the results from the children and young adults groups support the existence of a schema integration mechanism with better memory for expected endings than neutrals. This result is akin to the standard memory congruency effect and reflects the facilitation of the encoding of information when it is congruent with previous knowledge [5,11,16–18,40]. However, we did not observe the hypothesized absolute strengthening of this mechanism with age. In contrast to children, young adults showed a diminished quadratic component, with this reduction attributable to comparable memory for expected than for neutral endings. This result is paralleled in the older adults’ group, which despite showing increased memory for expected events, did not show a relative strengthening of this schema integration mechanism compared with young adults.

This unforeseen finding ought to be interpreted in the light of two considerations. First, the schema being unravelled in the task may have been too simplistic, resulting in negligible benefits from integrating elements into the well-consolidated schemas of the adults groups. A more complex schema might have provided enough room to significantly impact memory performance. Second, there was limited variability in the expected endings as the fulfilment of the prediction consistently led to the same outcome (i.e. the object being received by the second character). The uniform nature of these endings might have failed to engage the young participants’ memory systems as effectively as more varied or complex scenarios would have.

On the other hand, children and young adults, but not older adults, exhibited better memory for unexpected endings than for neutral ones. The reliable PE-driven encoding enhancement found in children and young adults is in line with previous reports of such effect [5,12,19,39,45,46] and further supports the existence of this mechanism by extending it to a new experimental paradigm. Moreover, our results also support the hypothesized developmental trajectory of the PE-driven encoding mechanism. The lack of a PE-driven boost in older adults is compatible with a hippocampal-dependent encoding following PE [11] as the integrity of medial temporal structures has been shown to decline with healthy ageing [47–49].

## 6. Conclusion

The results from the four memory tasks reflect a developmental profile characterized by a change in the relative contributions of the mechanisms within the age groups, whereas PE-driven encoding is prioritized during childhood, schema integration plays a larger role in older adulthood. Our results validate a novel approach to studying these two mechanisms with a paradigm that (i) includes stimuli suitable for testing across age groups; (ii) enables balanced frequency of the expectation matches and mismatches; and (iii) includes the temporal unfolding of events with careful control of the timing and amount of the information delivered.

Nevertheless, several questions remain open for future research. For instance, although evidence for the dissociation between the two mechanisms is gradually accumulating, we still do not know which of the constraints determine whether it is possible to measure the effects of one mechanism or the other. While the requirement of a neutral condition is a promising avenue [5,11], finding a perfectly balanced neutral condition is currently one of the major challenges. For instance, our paradigm, which exploits a common prediction with different outcomes, uses unfinished endings to avoid the prediction (mis)match. In doing so, our neutral condition prevents closure of the event which is known to impact memory formation [50,51]. In fact, this manipulation has been used previously to induce PE and to contrast it with prediction matches [27,52], thus capturing the left side of our *U*-shape pattern. An alternative possibility to create a neutral condition would be to vary the strength of the prediction while keeping the outcome constant. This approach has also been used to render different levels of PE to explore their impact on memory formation [33,38]. The extent to which the PEs obtained from these two approaches might render differential effects on encoding has not been comprehensibly explored and merits conceptual and experimental exploration.

Another factor provides further critical insights into the relationship between prediction and memory involves the operations that participants are carrying out during encoding [37,38,53]. In our task, participants performed a task which was directly related to the outcome of the prediction (i.e. they reported whether the object reached the second character). As such, affirmative responses were more frequent in the expected condition than in the other two. Previous studies have shown that affirmative responses can lead to advantages in encoding when compared with negative responses [54]. This affirmation-driven enhancement could thus underly schema-integration effects when a prediction-dependent response is required [55]. Future studies explicitly contrasting prediction-dependent with prediction-orthogonal tasks are required to further elucidate this relation.

The diversity of experimental approaches and the subsequent mixed pattern of findings in the literature [5,19,33,45,46,56] is still to be reconciled under a common framework. Computational modelling approaches hold a great potential in enabling an operationalization of these constraints [38,55] and in the delineation of the optimal situations in which schemas can be efficiently exploited [21]. Similarly, standardized definitions of the types of predictions researchers are using [1,57] can further clarify the boundary condition of these effects by isolating them from neighbouring ones [13,14].

The differential impact of expectedness across memory measures lends support to the existence of two separate mechanisms underlying episodic memory encoding. This dissociation is further supported by the distinct patterns observed across the lifespan. These different mechanisms are a core component of the dynamic interplay between the anticipation of future states and memory formation. Our study underscores the complexity of episodic memories, highlighting how different mechanisms come into play at various stages of life. These findings open up avenues for further research into the bidirectional relationship between memory formation and predictions.

## Data Availability

All data, materials and code are available from Zenodo at [58]. Supplementary material is available online [59].
